# Chronic social stress alters protein metabolism in juvenile rainbow trout, *Oncorhynchus mykiss*

**DOI:** 10.1007/s00360-021-01340-6

**Published:** 2021-03-12

**Authors:** Roxanne J. Saulnier, Carol Best, Daniel J. Kostyniuk, Kathleen M. Gilmour, Simon G. Lamarre

**Affiliations:** 1 Département de Biologie, Université de Moncton, Moncton, NB E1A 3E9 Canada; 2 Department of Biology, University of Ottawa, Ottawa, ON K1N 6N5 Canada

**Keywords:** Salmonid, Social hierarchy, Cortisol, Fractional rate of protein synthesis, Ubiquitin proteasome pathway, Autophagy lysosomal system

## Abstract

When confined in pairs, juvenile rainbow trout (*Oncorhynchus mykiss*) form dominance hierarchies in which subordinate fish exhibit characteristic physiological changes including reduced growth rates and chronically elevated plasma cortisol concentrations. We hypothesized that alterations in protein metabolism contribute to the reduced growth rate of socially stressed trout, and predicted that subordinate trout would exhibit reduced rates of protein synthesis coupled with increases in protein degradation. Protein metabolism was assessed in dominant and subordinate fish after 4 days of social interaction, and in fish that were separated after 4 days of interaction for a 4 days recovery period, to determine whether effects on protein metabolism recovered when social stress was alleviated. Protein metabolism was assessed in liver and white muscle by measuring the fractional rate of protein synthesis and markers of protein degradation. In the white muscle of subordinate fish, protein synthesis was inhibited and activities of the ubiquitin-proteasome pathway (UPP) and the autophagy lysosomal system (ALS) were elevated. By contrast, the liver of subordinate fish exhibited increased rates of protein synthesis and activation of the ALS. When allowed to recover from chronic social stress for 4 days, differences in protein metabolism observed in white muscle of subordinate fish during the interaction period disappeared. In liver, protein synthesis returned to baseline levels during recovery from social stress, but markers of protein degradation did not. Collectively, these data support the hypothesis that inhibition of muscle protein synthesis coupled with increases in muscle protein breakdown contribute to the reduced growth rates of subordinate rainbow trout.

**Supplementary Information:**

The online version contains supplementary material available at 10.1007/s00360-021-01340-6.

## Introduction

In fishes as in mammals, growth suppression is a widely observed feature of chronic stress (Sadoul and Vijayan 2016). For example, reduced growth rates are observed in fish subjected to prolonged crowding (Trenzado et al. 2006), poor water quality (Schram et al. 2009), or repeated handling, chasing and crowding (Barton et al. 1987; McCormick et al. 1998). Chronic social stress also suppresses growth in fishes (Metcalfe 1986; Abbott and Dill 1989; Sloman et al. 2000b; Buston 2003; Wong et al. 2007; Heg et al. 2004), and social subordination in juvenile salmonid fish has become a useful model in which to probe mechanisms underlying the effects of chronic stress. Linear dominance hierarchies form among juvenile salmonid fish through competition for feeding territories (Elliott 1990). Whereas the dominant fish within these hierarchies monopolize resources such as food and display high levels of aggression towards subordinate fish (Abbott and Dill 1985; Metcalfe 1986), behaviours such as feeding, activity and aggression are inhibited in subordinate fish (Abbott and Dill 1985, 1989; Adams et al. 1998; DiBattista et al. 2006). In addition to reduced growth rates (Metcalfe 1986; Abbott and Dill 1989; Sloman et al. 2000b); reviewed by (Gilmour et al. 2005), the physiological phenotype of subordinate fish includes chronic elevation of the glucocorticoid stress hormone cortisol (Ejike and Schreck 1980; Pottinger and Pickering 1992; Øverli et al. 1999; Sloman et al. 2000a); reviewed by (Gilmour et al. 2005; Sørensen et al. 2014).

Growth in fishes is determined by a variety of factors, including food intake, nutrient assimilation, and energy allocation to maintenance versus structural growth, all of which may be impacted by chronic stress (Sadoul and Vijayan 2016). For example, both appetite suppression and exclusion from food resources by dominant individuals reduce food intake in subordinate fish (DiBattista et al. 2006; Adams et al. 1998; Metcalfe et al. 1989). Digestive function is altered in subordinate fish (Peters 1982; Olsen and Ringo 1999; DiBattista et al. 2006), and liver metabolism is adjusted to favour the breakdown and metabolic use of glycogen and lipid reserves (DiBattista et al. 2006; Gilmour et al. 2012; Kostyniuk et al. 2018, 2019). In addition, standard metabolic rate increases in response to chronic social stress, indicating increased allocation of energy to maintenance (Sloman et al. 2000c). Although the lower growth rates of subordinate salmonids reflect the impact of chronic social stress on all of these factors, the most direct indicator of structural growth in fishes may be muscle protein growth (Sadoul and Vijayan 2016). Protein growth represents the balance between protein synthesis and protein degradation (Fraser and Rogers 2007; Johnston et al. 2011). Indices of protein degradation, such as plasma amino acid or ammonia concentrations (Haller and Wittenberger 1988; Vijayan et al. 1997; DiBattista et al. 2006), or molecular markers of proteolysis (Valenzuela et al. 2018), have been measured in some studies of chronic stress, but data are sparse. These effects of chronic stress are likely linked with the effects of cortisol on intermediary metabolism (reviewed in Van der Boon et al. (1991). However, and to our knowledge, the impact of chronic social stress on rates of protein synthesis has not yet been assessed.

The goal of the present study was to address this knowledge gap by evaluating the effects of chronic social stress on rates of protein synthesis and markers of protein degradation. Dominant and subordinate rainbow trout (*Oncorhynchus mykiss*) were compared after 4 days of social interaction, when subordinate fish are experiencing the behavioural (e.g. aggression) and physiological effects (e.g. elevated cortisol levels) of chronic social stress, and 4 days after separation of the dominant and subordinate members of a pair by placing an opaque partition into the tank. Culbert and Gilmour (2016) reported that food intake and growth remained suppressed in ‘recovering’ subordinates, but cortisol levels returned to normal baseline values. Therefore, this recovery protocol provides insight into the putative role played by cortisol in suppressing growth during chronic social stress. We measured fractional rates of protein synthesis in white muscle, which accounts for over half of the body mass in fishes (Mommsen et al. 1999; Sadoul and Vijayan 2016), and in the liver, where effects of chronic social stress on intermediary metabolism have been reported (DiBattista et al. 2006; Gilmour et al. 2012; Kostyniuk et al. 2018, 2019). Markers of protein degradation were assessed in these same two tissues, focusing on the ubiquitin–proteasome pathway (UPP) and the autophagic-lysosomal system (ALS) as the two proteolytic pathways that appear to play the largest roles in protein degradation in fish myocytes (Seiliez et al. 2014). Based on the hypothesis that reduced growth rates in subordinate rainbow trout are caused by decreases in protein accretion, we predicted that fractional rates of protein synthesis would be lower, and markers of protein degradation would be higher in subordinate fish when compared to their dominant counterparts.

## Materials and methods

### Experimental animals

Juvenile rainbow trout (mass = 111.4 ± 3.4 g; mean ± SEM, *N* = 67), were purchased from Linwood Acres Trout Farm (Campbellcroft, Ontario) and held at the University of Ottawa in 1275 L fibreglass tanks supplied with flowing, aerated, dechloraminated city of Ottawa tap water at a temperature of 13 °C. Fish were exposed to a photoperiod of 12L:12D and scatter-fed commercial trout pellets at a ration of 0.5% body mass per day, except for the 2 days prior to experimentation when food was withheld to eliminate effects of food intake on protein metabolism. The holding conditions (use of scatter-feeding, homogeneous tanks with a mild current) minimized the formation of social hierarchies and were maintained for at least 2 weeks prior to experimentation. Experimental protocols were approved by the animal care committees of the Université de Moncton and the University of Ottawa and followed the guidelines of the Canadian Council on Animal Care for the use of animals in research and teaching.

### Methods validation

In preliminary experiments, an appropriate tracer incorporation period for the flooding dose method of measuring fractional rates of protein synthesis (Lamarre et al. 2015) was determined for rainbow trout held at 13 °C. Fish (*N* = 15) were lightly anaesthetized in a solution of benzocaine (50 mg L^−1^ ethyl-*p*-aminobenzoate; Sigma-Aldrich, Oakville, ON, Canada), and mass and fork length were measured. Fish were placed in 4 L individual Plexiglass chambers supplied with flowing, aerated 13 °C water. Following an overnight recovery period, unanesthetized fish were given an intra-peritoneal injection of 150 mM phenylalanine containing 50% ring-D_5_ l-phenylalanine (D_5_-PHE, 98%; Cambridge Isotope Laboratories, Andover, MA, USA) in distilled water at a dosage of 1 mL 100 g^−1^. After 0, 60, 120, 240 or 360 min, fish (*N* = 3 at each time) were rapidly euthanized via immersion in a terminal anaesthetic solution (500 mg L^−1^ ethyl-*p*-aminobenzoate). The peritoneal cavity was immediately exposed and rinsed with distilled water to wash away unabsorbed tracer. Liver and white muscle samples were collected, flash frozen in liquid nitrogen, and stored at – 80 °C until analysis.

### Experimental protocol

Protein metabolism was compared between dominant and subordinate trout after 4 days of social interaction (*N* = 6 pairs), and after a 4 days interaction period followed by 4 days of recovery (*N* = 6 pairs; as in Culbert and Gilmour (2016)). Sham-treated fish (*N* = 6 at each sampling time) were included in the experimental design as a control for the effects of handling associated with establishing and monitoring social hierarchies; these fish were sampled after 4 or 8 days in the same way as paired fish but were always held without a conspecific.

Fish were lightly anaesthetized in a solution of benzocaine (50 mg L^−1^ ethyl-*p*-aminobenzoate), initial mass and fork length were measured, and any fin damage was noted (Moutou et al. 1998). Fish were size-matched according to fork length (Abbott and Dill 1985) with differences not exceeding 5% (fork length difference averaged 3.7 ± 0.8 mm or 1.7 ± 0.4% of fork length; *N* = 12 pairs). The left or right pectoral fin was clipped for identification of individuals within a pair. Pairs were placed in 40 L flow-through Plexiglass tanks supplied with flowing, aerated water at 13 °C, with the members of the pair separated by an opaque, perforated divider. Following an overnight recovery period, the divider was removed, and the fish were allowed to interact for 4 days. Behavioural observations were carried out once in the morning between 9 and 11 am, and once in the afternoon between 2 and 4 pm (for 5 min each time) to determine the social status of each fish. Behaviours were scored using a system that awards more points for more dominant behaviours such as patrolling the tank, taking a food item, or carrying out aggressive acts (as previously described by Sloman et al. 2001). At the end of each observation period, pairs (and shams) were offered one pellet of food but were not otherwise fed for the duration of the experiment. The potential effects of feeding (a maximum of two pellets per day) on protein metabolism were later assessed using a second set of sham-treated fish (*N* = 5) that were exposed to the same protocol but not offered food. The mean scores over the observation period for patrolling, taking a food item and aggression were compiled with fin damage scores via principal components analysis (Minitab, v16) to yield a behaviour score for each fish. The individual within a pair with the higher behaviour score was assigned dominant status. Pairs with similar behaviour scores (a difference of < 0.5) were excluded from further analysis (*N* = 4 pairs) for having failed to form a hierarchy. At the end of the 4 days interaction period, protein synthesis was evaluated for half of the pairs (*N* = 6 pairs) and sham-treated fish (*N* = 6 shams). For the remaining pairs and shams (*N* = 6 pairs, *N* = 6 shams), the opaque divider was replaced to separate the members of the pair for a 4 days recovery period prior to the measurement of protein synthesis. Using the same behaviour tanks, Culbert and Gilmour (2016) showed that cortisol levels in the subordinate fish rapidly recover once it has been separated from the dominant by the divider.

To measure fractional rates of protein synthesis, fish received an intra-peritoneal injection of tracer phenylalanine solution as described above. Following a 2-h incorporation period (determined experimentally, see above), fish were rapidly euthanized via immersion in a terminal anaesthetic solution (500 mg L^−1^ ethyl-*p*-aminobenzoate). Fin damage was scored, and mass and fork length were measured. A blood sample (~ 0.5 mL) was collected via caudal venipuncture with an EDTA-coated syringe and centrifuged at 13,000*g* for 3 min. The plasma was flash frozen in liquid nitrogen for later analysis of plasma cortisol concentration. Plasma cortisol levels were measured via radioimmunoassay (RIA; MP Biomedical), with a detection limit of 0.17 µg dL^−1^. Intra-assay variation (% CV) was 7.9% and inter-assay variation was 12.2%. White muscle and liver tissue were collected, immediately flash frozen in liquid nitrogen, and stored at – 80 °C until analysis.

### Analytical techniques

#### Measurement of fractional rates of protein synthesis

The fractional rate of protein synthesis was measured using a modified version of the flooding dose technique (Lamarre et al. 2015). Approximately 75 mg of tissue were homogenized using a sonicating homogenizer (Q55 Sonicator, Qsonica, Newtown, CT, USA) in 1 mL of 0.2 M perchloric acid (PCA). Samples were centrifuged at 12,000*g* for 5 min at 4 °C to separate the free amino acid pool from the protein pool. The supernatant, containing the free amino acids, was saved and stored at − 20 °C for later analysis. The protein pellet was washed in 1 mL of 0.2 M PCA and centrifuged (5 min at 12,000*g* at 4 °C) three additional times. Protein pellets were washed a fourth time in 1 mL of acetone to remove lipids and were then hydrolyzed in 6 M hydrochloric acid at 110 °C for 18 h. Phenylalanine was extracted from the hydrolyzed protein-pool and the free-pool samples using solid-phase extraction (Bond-Elut-C18, 100 mg, 1 mL; Varian, Palo Alto, CA, USA) as described in Cassidy et al (2016). The extracted amino acids were dried at 110 °C for ~ 2 h before being derivatized via an alkylation procedure using pentafluorobenzyl bromide (PFBBr) as previously described (Lamarre et al. 2015). The D_5_-PHE-specific enrichment of the free-pool (*S*_a_) and protein-pool (*S*_b_) samples was determined by gas chromatography (model 7890B; Agilent Technologies, Santa Clara, CA, USA) interfaced with a single quadrupole inert mass selective detector (MSD, model 5977B). The fractional rate of protein synthesis was calculated as:

$$ K_{{\text{s}}} (\% \;{\text{day}}^{ - 1} ) = \frac{{S_{{\text{b}}} }}{{S_{{\text{a}}} }} \times \frac{1440}{t} \times 100 $$

where *S*_b_ is the enrichment of the protein-pool [*S*_b_ = D_5_-PHE /(PHE + D_5_-PHE)] and *S*_a_ is the enrichment of the free amino acid pool [*S*_a_= D_5_-PHE /(PHE + D_5_-PHE)], *t* is the tracer incorporation time, and 1440 is the conversion from minutes to days (Garlick et al. 1983; Lamarre et al. 2015).

#### Measurement of transcript abundance by real-time RT-PCR

Transcript abundances of genes coding for protein degradation enzymes were assessed by real-time RT-PCR. Total RNA was extracted from liver and white muscle using the TRI Reagent® protocol (Sigma) according to the manufacturer’s instructions. RNA extracts were quantified using a NanoVue Plus (GE Healthcare Limited, Little Chalfont, Bucks, UK) and diluted to 200 µg mL^−1^. Next, 50 μg of total RNA was treated with DNase (DNA-free kit; Life Technologies, Carlsbad, CA, USA) and 1.5 µg of DNase-treated RNA was reverse transcribed into cDNA using qScript cDNA SuperMix (Quanta Bioscience, Gaithersburg, MD, USA) according to the manufacturer’s instructions. Real-time RT-PCR primers (Table 1) for cathepsin D, cathepsin L, muscle atrophy F-box (MAFbx), and muscle RING-finger protein-1 (MuRF1) and the housekeeping gene, β-actin, were designed using Primer 3 (http://bioinfo.ut.ee/primer3-0.4.0/), tested using OligoAnalyzer 3.1 (https://www.idtdna.com/calc/analyzer) to avoid hairpins, homo- and cross-dimers, and validated via sequencing at the Centre de Recherche du CHUL at the Université Laval (Québec, QC, Canada). Sequences were subsequently verified via BLAST (NCBI).

**Table 1:** Gene-specific primers used for real-time RT-PCR

Gene	Primer sequence (5′ to 3′)	Amplicon length (bp)	Annealing temperature (ºC)	Liver—Efficiency (%)	White muscle—Efficiency (%)	Accession #
*mafbx*	F: CACACTGCCACATCCTCTTC	70	59.26	105	96	NC_027301.1
	R: CAGCAGCTCTCTGTGTTGTTG					
*murf1*	F: CTCCATGTGCAAAGTGTTCG	155	60.30	–	101	NM_001279122.1
	R: TGTCCTCCATCTGAGCCATC					
*cathepsin L*	F: GTCAAGGACCAGGGATCATGT	120	59.44	90	110	NM_001146546.1
	R: GTCCACCAGGTTCTGTTCAC					
*cathepsin D*	F: ATCTGCCTGAGTGGCTTCAT	124	59.80	93	109	NM_001124711.1
	R: GTTGTCACGGTCGAACACAG					
*ß-actin*	F: CATCAGGGAGTGATGGTTGG	79	61.30	95	103	NM_001123525.1
	R: TCAGGATACCCCTCTTGCTC					

*mafbx*, muscle atrophy F-box; *murf1*, muscle RING-finger protein-1

Semi-quantitative real-time RT-PCR was performed in triplicate for each cDNA sample on a CFX Connect Real-Time PCR Detection System (Bio-Rad, Hercules, CA, USA). Reactions consisted of 5 µL of Sso advanced Universal SYBR Green (Bio-Rad), 4 µL of cDNA template, 400 nM of forward and reverse primers, and DEPC water to 10 µL. Primer amplification efficiencies were measured using tenfold serial dilutions of pooled samples and ranged from 90 to 110% (Table 1). The reaction conditions included polymerase activation and DNA denaturation steps of 10 min at 95 °C, 39 amplification cycles with denaturation of 15 s at 95 °C and annealing/extension of 30 s at 60 °C, and finished with a melt-curve analysis from 65 to 95 °C with a 0.5 °C increment for 5 s per step. Threshold lines were verified to ensure that they intersected with the amplification lines in the linear portion of the curve and melt curves were analyzed to confirm amplification of a single product. Cycles to threshold (Ct) were recorded and relative expression was calculated using the Livak method (Livak and Schmittgen 2001). Values were expressed as fold change relative to the average of sham-treated fish using the reference gene ß-actin. Reference gene stability across status and interaction period was confirmed via two-way analysis of variance (ANOVA) on reference gene Cts.

#### Measurement of 20S proteasome activity

The maximal chymotrypsin-like activity of the 20S proteasome was measured enzymatically (modified from Shibatani and Ward (1995). Tissues were homogenized in five volumes of lysis buffer (50 mM Tris, 0.1 mM EDTA, 0.007% mercaptoethanol, pH = 8) using a sonicating homogenizer (Q55 Sonicator, Qsonica), then centrifuged at 20,000 *g* for 20 min at 4 °C. The protein concentration of the supernatant was measured with a Bradford protein assay kit (Bio-Rad). Samples were assayed in quadruplicate in a black-bottomed 96-well plate. Each well contained 10 µL of sample homogenate and 10 µL of LLVY-AMC (400 μM in Tris buffer; P802-0005, Enzo Life Sciences, Burlington, ON, Canada). The fourth replicate also contained 10 µL of a specific inhibitor of the 20S proteasome (ZLLL-CHO; PI102-0005, Enzo Life Sciences). Finally, 100 µL of assay buffer (100 mM Tris, 0.0475% SDS, pH = 8) was added to all wells and maximal 20S proteasome activity was determined by measuring fluorescence at excitation/emission wavelengths of 370/430 nm in a multimode plate reader (Victor *X*3, Perkin Elmer, Waltham, MA, USA). The activity of the 20S proteasome was expressed in arbitrary fluorescence units per minute, per μg of protein.

#### Levels of polyubiquitinated proteins

Dot blot analyses carried out on aliquots of the tissue homogenates generated for 20S proteasome activity (see above) were used to compare levels of polyubiquitinated proteins. Samples were spotted in triplicate on a nitrocellulose membrane at concentrations of 3 and 1.5 μg protein, for white muscle and liver, respectively. The membrane was blocked for 1 h with 5% bovine serum albumin (BSA) and then incubated for 1 h with an anti-poly-ubiquitin antibody (catalogue # ab190061, Abcam, Cambridge, MA, USA) specific for the detection of K48-linked polyubiquitin chains. Dots were imaged via enhanced chemiluminescence (ECL), using a horseradish peroxidase (HPR) conjugated antibody (catalogue # ab97023, Abcam) and Chemidoc (Bio-Rad). Spot intensity was quantified using Image Lab 5.1 (Bio-Rad). Dot densities were normalized to protein concentration.

### Statistical analyses

Values are expressed as means ± SEM. Specific growth rate (SGR) was calculated as [ln(*m*_Final_) − ln(*m*_Initial_)] × *100/D*, where *m* is the mass of the fish in grams and *D* is the number of days that elapsed between measurements of mass. Linear regression was used to determine an appropriate tracer incorporation period. One- or two-way ANOVA with Holm-Sidak multiple comparisons tests was used to assess the effects social status (dominant, subordinate, sham) and treatment group (interaction versus recovery) on all variables. Two-tailed unpaired Student’s *t*-tests were used to compare shams with fasted shams, to assess the effects of feeding on all variables (see supplementary material). Data were transformed or equivalent non-parametric tests were used when assumptions of normality or equal variance were not met. The fiducial limit of significance was 0.05 and statistical analyses were carried out in Prism v7 (GraphPad Software Inc., La Jolla, CA, USA) or Sigmaplot v13 (Systat Software Inc., Chicago, IL, USA).

## Results

**Fig. 1:**
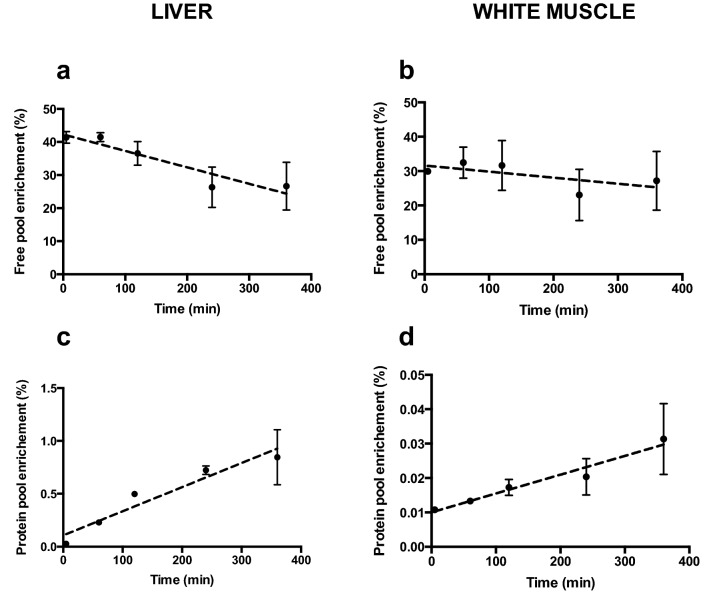
Specific enrichment (%) of the phenylalanine free-pool of liver (**a**) and white muscle (**b**), and the protein pool of liver (**c**) and white muscle (**d**), in relation to time (min) post injection of the tracer in rainbow trout (*Oncorhynchus mykiss)*. Values are means ± SEM with *N* = 3 per time point. The equations for the regression lines for free-pool enrichment (*S*_a_) are **a** *S*_a_ (%) = − 0.04923 × time + 42.16, *R*^2^ = 0.46, *P* = 0.0057, and **b** *S*_a_ (%) = − 0.01736 × time + 31.56, *R*^2^ = 0.05, *P* = 0.4037. The equations for the regression lines for protein pool enrichment (*S*_b_) are **c** *S*_b_ (%) = 0.002265 × time + 0.1114, *R*^2^ = 0.71, *P* < 0.0001, and **d** *S*_b_ (%) = 5.421e-005 × time + 0.01016, *R*^2^ = 0.45, *P* = 0.0058

A time trial was carried out to determine an appropriate tracer incorporation period. The flooding dose technique is based on four assumptions (24) that must be met for this method of measuring the fractional rate of protein synthesis to be valid: (1) the isotope enrichment must be detectable within a short period of time; (2) the free-pool enrichment must remain stable over time; (3) the protein-pool enrichment must increase in a linear fashion over time; and (4) the isotope tracer used must not alter overall protein metabolism (Fraser and Rogers 2007; Garlick et al. 1980; Lamarre et al. 2015). Following injection of the tracer, enrichment of the free-phenylalanine pool (*S*_a_) quickly rose, to 41.4 ± 1.8% and 29.9 ± 1.0% in liver and white muscle, respectively (Fig. 1a, b). Although it decreased slightly over six hour period, the enrichment remained elevated and stable during the first two hours in muscle and liver (ANOVA, *P* = 0.939 and *P* = 0.869, respectively). The incorporation of the tracer into the protein pool (*S*_b_) increased in a linear fashion over time in both liver and white muscle and significant enrichment was detected after 1 h of incorporation (Fig. 1c, d). Based on these results, an incorporation period of 2 h was deemed to be appropriate.

Rainbow trout held in pairs for 4 days formed social hierarchies as evidenced by low behaviour scores in subordinate relative to dominant fish (Table 2; Student’s *t* tests, *P* < 0.001 for interaction pairs and recovery pairs). Subordinate fish exhibited significantly higher plasma cortisol levels (ANOVA on log-transformed data, *P* = 0.009) than dominant or sham-treated fish after 4 days of interaction and no food intake. Specific growth rates were significantly lower in subordinate than dominant fish, with neither differing from sham-treated fish (ANOVA, *P* = 0.023). These differences were alleviated by physically separating dominant and subordinate fish (by replacement of the divider in the tank) for 4 days of recovery, such that after a recovery period, none of the cortisol levels (ANOVA on log-transformed data, *P* = 0.305), SGR (ANOVA, *P* = 0.083) or food intake (ANOVA, *P* = 0.102) differed among dominant, subordinate and sham-treated fish (Table 2).

**Table 2:** Behaviour scores, plasma cortisol concentrations, specific growth rates and feeding scores of dominant, subordinate and sham-treated rainbow trout (*Oncorhynchus mykiss)* at the end of a 4-day interaction period (‘Interaction’) or following 4 days of interaction and a 4-day recovery period (‘Recovery’)

	Interaction			Recovery		
	Sham	Dominant	Subordinate	Sham	Dominant	Subordinate
Behaviour score during interaction	–	1.9 ± 0.2^a^	− 1.9 ± 0.3^b^	–	1.5 ± 0.3^A^	− 1.5 ± 0.3^B^
Plasma [cortisol] (ng mL^−1^)	20.9 ± 9.3^a^	14.5 ± 5.0^a^	202.6 ± 64.9^b^	34.6 ± 20.8	25.9 ± 6.1	48.8 ± 12.3
SGR (% day^−1^)	–1.27 ± 0.25^ab^	− 0.78 ± 0.20^a^	– 1.80 ± 0.22^b^	− 0.52 ± 0.10	− 0.52 ± 0.11	− 0.85 ± 0.12
Feeding score during interaction	–	1.0 ± 0.0	0.0 ± 0.0	0.6 ± 0.2^AB^	0.9 ± 0.1^A^	0.1 ± 0.1^B^
Feeding score during recovery	–	–	–	0.7 ± 0.2	0.9 ± 0.1	0.4 ± 0.2

Feeding was scored as 1 for taking the single pellet that was offered or 0 if the pellet was not ingested. Scores for an individual fish were averaged over all observation periods during the 4 days interaction period or the 4 days recovery period. Values are means ± SEM, *N* = 6 for all groups except dominant fish sampled at 4 days of interaction, where *N* = 5. Within a treatment (interaction or recovery), groups that share a letter are not significantly different from one another (see text for details). *SGR* specific growth rate

**Fig. 2:**
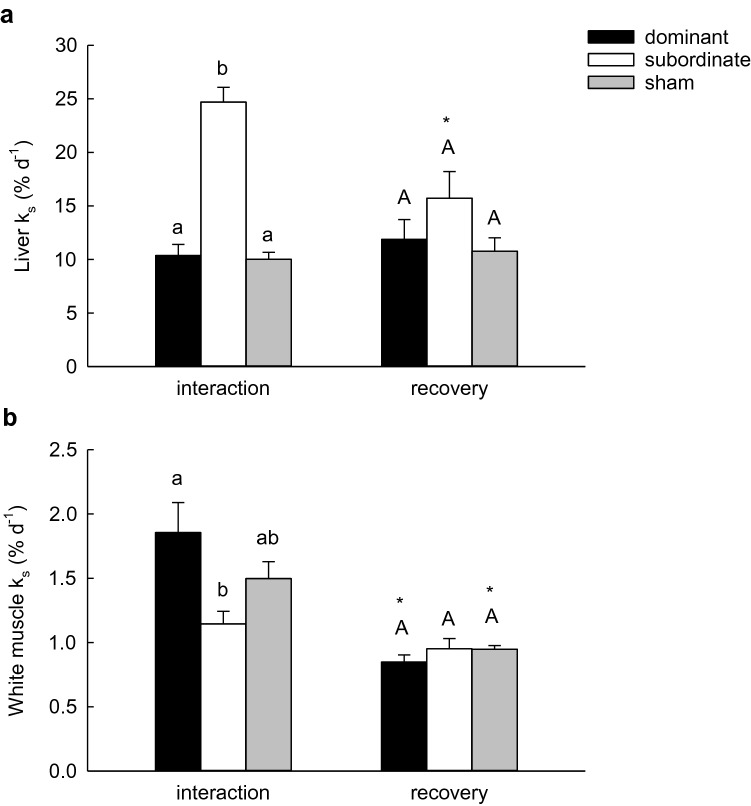
Fractional rates of protein synthesis in the liver (**a**) and white muscle (**b**) of dominant, subordinate and sham-treated rainbow trout (*Oncorhynchus mykiss)* at 4 days of interaction period or following 4 days of recovery from a 4-days period of social interaction. Values are means + SEM with *N* = 5 for dominant fish in the interaction treatment, and *N* = 6 for all other groups. Within a treatment (interaction or recovery), groups that share a letter are not significantly different from one another. An asterisk indicates a significant difference between interaction and recovery values within a social status category (2-way ANOVA, see text for details)

Fractional rates of protein synthesis (*k*_s_) were significantly affected by social status and treatment group (interaction or recovery). In liver, *k*_s_ was significantly higher in subordinate than dominant or sham fish after 4 days of interaction, but not after 4 days of recovery from social stress (Fig. 2a; 2-way ANOVA, *P*status × treatment = 0.004). By contrast, *k*_s_ in white muscle was significantly lower in subordinate than dominant fish with neither differing from sham-treated fish after 4 days of interaction (Fig. 2b; 2-way ANOVA on ranks, *P*status × treatment = 0.025). Again, following the 4 days recovery period, *k*_s_ did not differ among sham, dominant and subordinate fish. However, in dominant and sham-treated fish (but not subordinates), *k*_s_ was significantly lower in fish sampled after a recovery period than in fish sampled at 4 days of interaction.

**Fig. 3:**
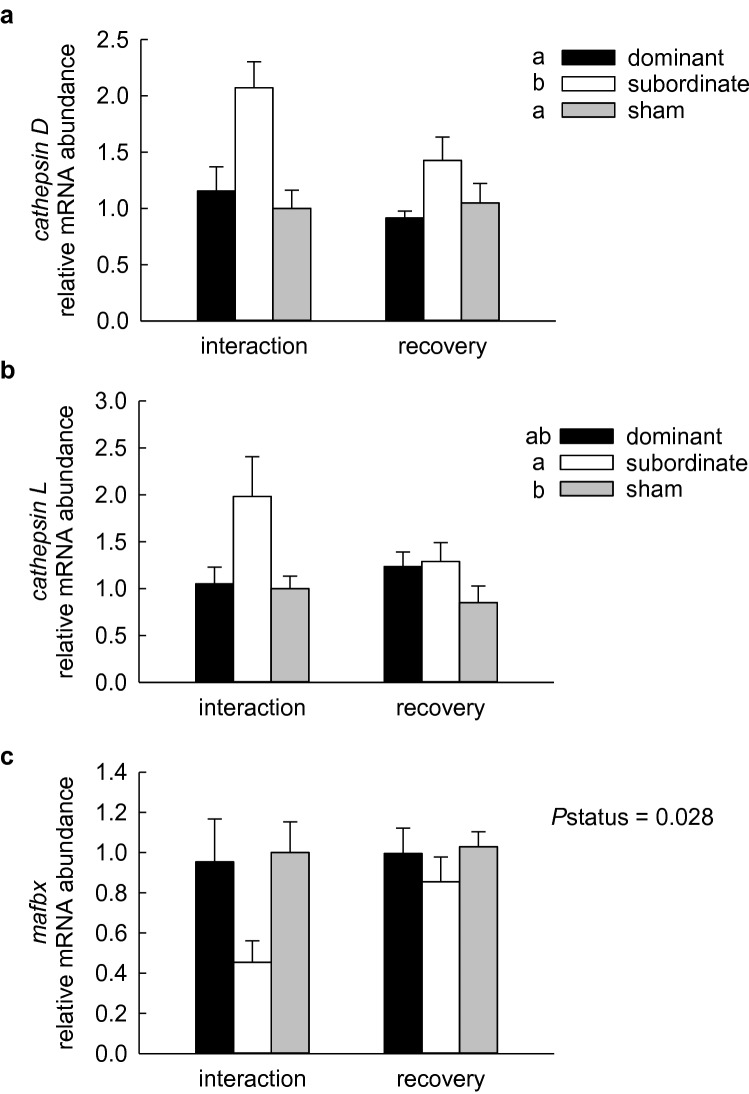
Relative mRNA abundance of *cathepsin D* (**a**), *cathepsin L* (**b**), and *mafbx* (**c**) in liver of dominant, subordinate and sham-treated rainbow trout (*Oncorhynchus mykiss)* at 4 days of social interaction or following 4 days of recovery from a 4-days interaction period. Values are expressed relative to the sham group sampled at 4 days of interaction. Values are means + SEM with *N* = 4–6 for all groups. Social status categories that share a letter are not significantly different from one another (2-way ANOVA, see text for details)

**Fig. 4:**
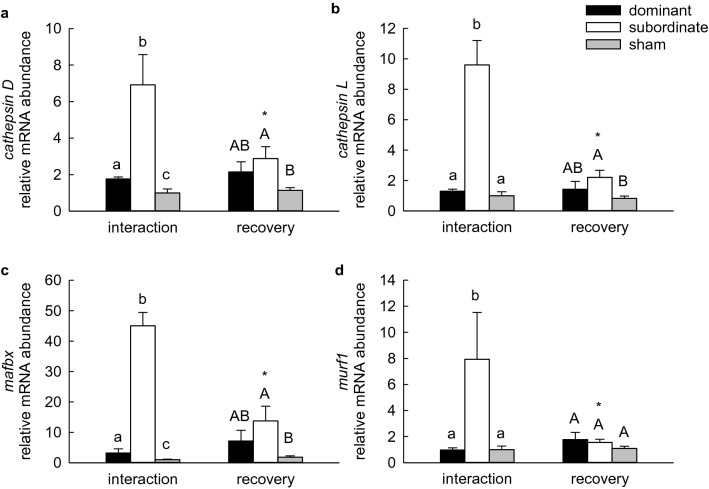
Relative mRNA abundance of *cathepsin D* (**a**), *cathepsin L* (**b**), *mafbx* (**c**) and *murf1* (**d**) in white muscle of dominant, subordinate and sham-treated rainbow trout (*Oncorhynchus mykiss*) at 4 days of social interaction or following 4 days of recovery from a 4-days interaction period. Values are expressed relative to the sham group sampled at 4 days of interaction. Values are means + SEM with *N* = 5–6 for all groups. Within a treatment (interaction or recovery), groups that share a letter are not significantly different from one another. An asterisk indicates a significant difference between interaction and recovery values within a social status category (2-way ANOVA, see text for details)

Several markers of protein degradation were evaluated, including transcript abundances of genes in the UPP and ALS, abundance of polyubiquitinated proteins, and enzyme activity of the 20S proteasome. In liver, transcript abundances of the lysosomal proteases *cathepsin D* and *cathepsin L* were significantly elevated in subordinate fish. Neither the effect of treatment group nor the interaction of social status and treatment group were significant (Fig. 3a, b; 2-way ANOVA, *P*status < 0.001, *P*treatment = 0.088, *P*status × treatment = 0.182 for *cathepsin D*; *P*status = 0.014, *P*treatment = 0.296, *P*status × treatment = 0.289 for log-transformed data for *cathepsin L*). A significant effect of social status was detected for transcript abundance of the ubiquitin ligase *mafbx*, again with no significant effect of treatment group or the interaction term (Fig. 3c; 2-way ANOVA, *P*status = 0.028, *P*treatment = 0.186, *P*status × treatment = 0.322). Although post hoc tests could not identify the source of the significant difference, values for subordinate fish appeared to be lower than those for dominant or sham-treated fish. In white muscle, transcript abundances of *cathepsin D*, *cathepsin L*, *mafbx* and *murf1*, another ubiquitin ligase, were significantly elevated in subordinate fish compared to dominants and shams at the end of the 4-days interaction period (Fig. 4; 2-way ANOVA on log-transformed data, *P*status × treatment = 0.039, 0.007, 0.004, 0.007 for *cathepsin D*, *cathepsin L*, *mafbx*, and *murf1*, respectively). In all cases, values for recovering subordinates were significantly lower than those at the end of the interaction period, whereas values for dominant and sham-treated fish did not differ between the interaction and recovery groups. Within the recovery treatment group, values for dominant and subordinate fish did not differ, although transcript abundances of *cathepsin D*, *cathepsin L* and *mafbx* were slightly but significantly higher in subordinate than sham-treated fish.

**Fig. 5:**
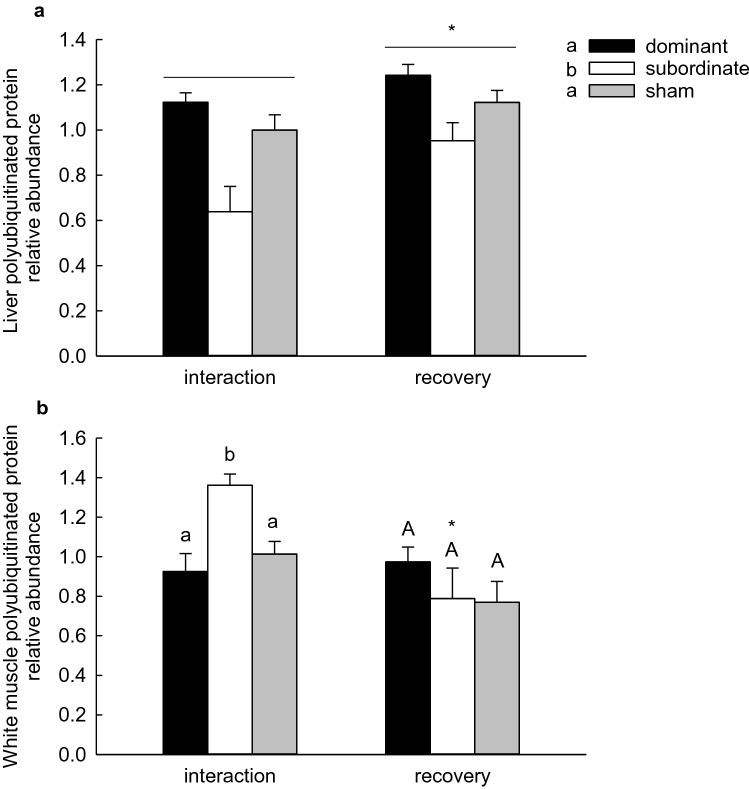
Relative abundance of polyubiquitinated proteins in the liver (**a**) and white muscle (**b**) of dominant, subordinate and sham-treated rainbow trout (*Oncorhynchus mykiss)* at 4 days of social interaction or following 4 days of recovery from a 4-days interaction period. Values are expressed relative to the sham group sampled at 4 days of interaction. Values are means + SEM with *N* = 5–6 for all groups. In panel **a**, social status categories that share a letter are not significantly different from one another and an asterisk indicates a significant difference between interaction and recovery values (2-way ANOVA, see text for details). In panel **b**, groups within a treatment (interaction or recovery) that share a letter are not significantly different from one another. An asterisk indicates a significant difference between interaction and recovery values within a social status category (2-way ANOVA, see text for details)

**Fig. 6:**
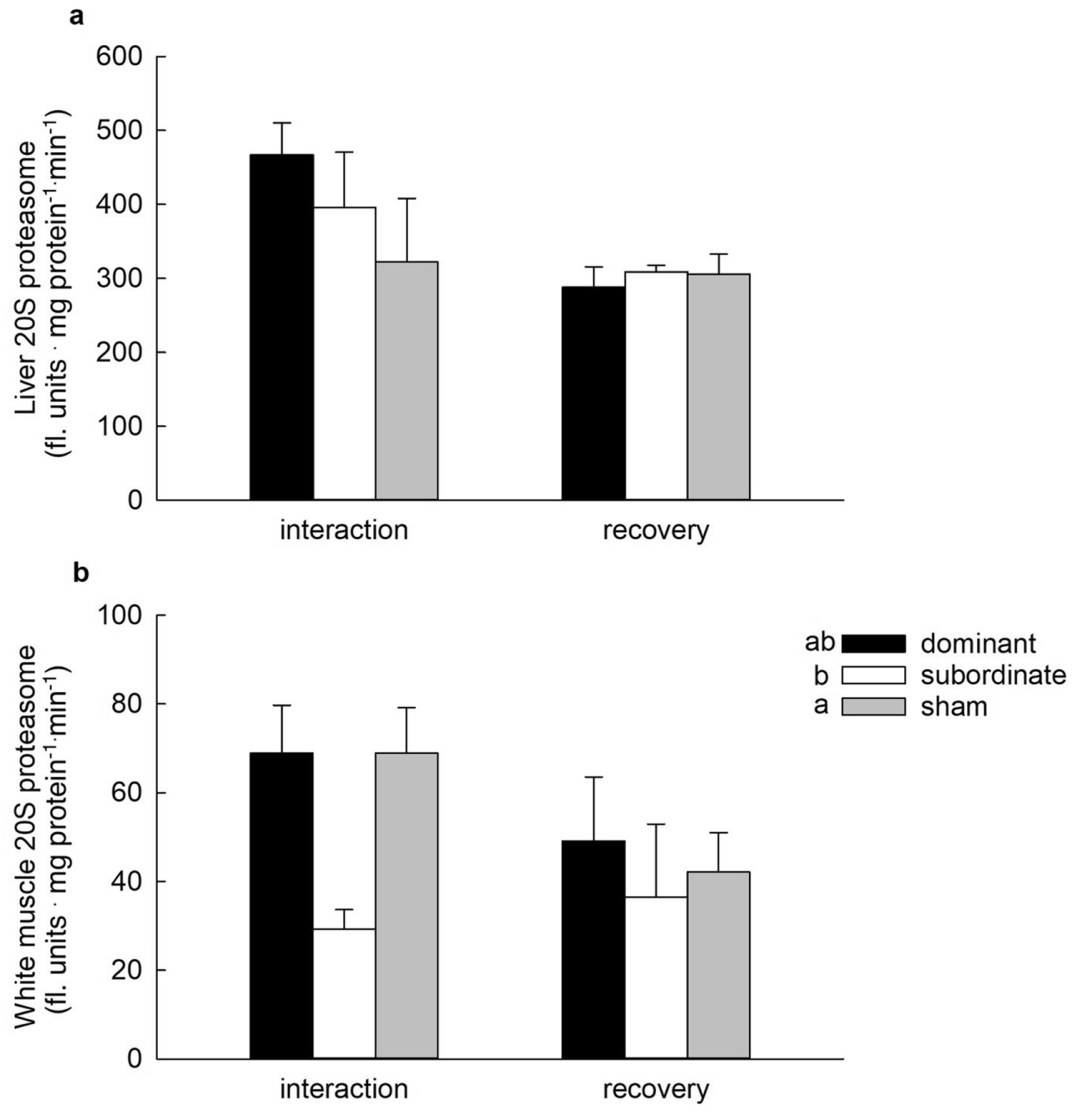
Maximal chymotrypsin-like activity of the 20S proteasome (20S proteasome) in the liver (**a**) and white muscle (**b**) of dominant, subordinate and sham-treated rainbow trout (*Oncorhynchus mykiss*) at 4 days of social interaction or following 4 days of recovery from a 4-days interaction period. Values are means + SEM with *N* = 4–6 for all groups. In panel **a**, no significant differences were detected (2-way ANOVA, see text for details). In panel **b**, social status categories that share a letter are not significantly different from one another (2-way ANOVA, see text for details)

To provide additional indicators of UPP activity, the abundance of polyubiquitinated proteins was assessed together with maximal chymotrypsin-like activity of the 20S proteasome. In liver, polyubiquitinated protein abundance was significantly higher in fish of the recovery group than in fish sampled after 4 days of interaction, and was significantly lower in subordinate fish than in sham or dominant fish, with no significant interaction between these factors (Fig. 5a; 2-way ANOVA, *P*status < 0.001, *P*treatment = 0.004, *P*status × treatment = 0.311). In white muscle, polyubiquitinated protein abundance was significantly higher in subordinate than dominant or sham fish after 4 days of interaction, but not after 4 days of recovery from social stress (Fig. 5b; 2-way ANOVA, *P*status = 0.178, *P*treatment = 0.004, *P*status × treatment = 0.016). No significant effect of social status or treatment group was detected for the maximal chymotrypsin-like activity of the 20S proteasome in liver (Fig. 6a; 2-way ANOVA on ranks, *P*status = 0.448, *P*treatment = 0.055, *P*status × treatment = 0.218). In white muscle, 20S proteasome activity was significantly lower in subordinate than sham fish, with neither differing from dominant fish, a pattern that was not affected by treatment group (Fig. 6b; 2-way ANOVA, *P*status = 0.033, *P*treatment = 0.081; *P*status × treatment = 0.781).

Although food generally was withheld to eliminate effects of food intake on protein metabolism, willingness to take food was used as an indicator of social status. The potential effects of this small amount of food intake (a maximum of two pellets per day) were assessed by comparing values in sham-treated fish with those for a separate group of fasted sham fish. No differences were detected between sham-treated fish and fasted shams (Supplementary material, Table S1).

## Discussion

As in previous studies (Abbott and Dill 1985; Culbert and Gilmour 2016; DiBattista et al. 2006; Sloman et al. 2001), holding juvenile rainbow trout in pairs for a period of four days resulted in behavioural differences that allowed dominant and subordinate individuals to be distinguished. Dominant and subordinate fish also differed physiologically, with subordinate fish exhibiting the lowered growth rates and elevated cortisol levels that have been reported previously (e.g. Abbott and Dill 1989; Culbert and Gilmour 2016; DiBattista et al. 2006; Sloman et al. 2001; Winberg and Lepage 1998). Collectively, these behavioural and physiological differences confirmed that dominant and subordinate phenotypes were obtained in the present study. Culbert and Gilmour (2016) reported that separation of dominant and subordinate fish in the observation tank using an opaque, perforated partition allowed subordinate fish to regain normal stress axis activity, although metabolic differences remained even after 4 days of recovery. A similar but stronger recovery was apparent after 4 days of separation in the present study, because plasma cortisol levels, growth rates, and feeding scores for recovering subordinates did not differ statistically from those of recovering dominant or sham-treated fish. The finding that preventing visual cues with a perforated barrier is enough to relieve the chronic social stress experienced by the subordinate fish supports the view that visual cues are more important than chemical cues in intra-specific communication in salmonid social hierarchies, as previously suggested (Höjesjö et al. 2015). Fish of all social and treatment categories exhibited negative growth rates because food was withheld throughout the study to avoid confounding effects of food intake on protein metabolism. An exception was made to offer one food pellet per observation period because monopolization of food resources is a strong indicator of dominant status (Abbott and Dill 1985; Metcalfe 1986). The comparison of sham-treated and fasted sham fish revealed no differences, indicating that the small amount of food intake associated with behavioural observations (a maximum of two pellets per day) was without measurable effect on protein metabolism. Based on these behavioural phenotypes, we predicted that the decreased growth rates of subordinate trout would reflect decreases in the rate of protein synthesis and/or increases in protein degradation.

In agreement with our prediction, the fractional rate of protein synthesis in the muscle of subordinate fish was 40% lower than that in dominant fish and 23% lower than that in sham-treated fish, although the latter difference failed to reach statistical significance. However, the fractional rate of protein synthesis in the liver of subordinate rainbow trout was double that of dominant and sham fish during the interaction period, with no differences among recovering subordinates, dominants and sham-treated fish. Although unexpected in the context of the low growth rates and high cortisol levels of subordinate trout, this elevated rate of protein synthesis is consistent with the substantial increase in P70S6K phosphorylation status previously reported in the liver of subordinate trout (Kostyniuk et al. 2018). Whether the elevated rate of protein synthesis reflects an increase in overall protein synthesis in liver tissue or an increase in the synthesis of a few specific proteins or protein families remains to be determined. This could be achieved using a proteomic approach as previously described (Doherty et al. 2013). Chronic social stress in rainbow trout alters hepatic carbohydrate and lipid metabolism to mobilize stored glycogen, enhance gluconeogenic potential, and increase ß-oxidation of free fatty acids (DiBattista et al. 2006; Gilmour et al. 2012; Kostyniuk et al. 2018). Increases in the transcript abundances of genes such as the gluconeogenic enzyme phosphoenolpyruvate carboxykinase (*pck1*), hormone-sensitive lipase (*hsl*), and the ß-oxidation marker carnitine palmityoltransferase 1A (*cpt1a*) suggest that these metabolic changes are achieved, at least in part, through increased synthesis of key proteins (Kostyniuk et al. 2018, 2019). In addition, social interactions increased the abundance of heat shock proteins (HSP) in the liver of rainbow trout (Currie et al. 2010). These responses may account for the elevated rate of protein synthesis in the liver of subordinate trout, but further investigation is needed, through approaches such as quantitative proteomics, to identify the proteins being synthesized.

The reduced rate of protein synthesis in the muscle of subordinate trout was accompanied by increases in markers of protein degradation, suggesting increased rates of muscle protein breakdown. The data suggested the activation of both the UPP and ALS. Transcript abundances of *mafbx* and *murf1*, genes coding for E3 ubiquitin ligases, which are responsible for the transfer of ubiquitin onto targeted proteins (Ardley and Robison 2005), were significantly higher in the muscle of subordinate fish, as was the relative abundance of polyubiquitinated proteins, providing support for increased muscle protein degradation via the ubiquitin–proteasome system. Lysosomal cathepsins are the primary drivers of protein degradation in fish muscle (Cassidy et al. 2016; Seiliez et al. 2014), and transcript abundances of the proteases *cathepsin D* and *cathepsin L* were significantly elevated in the white muscle of subordinate trout. All of these differences were eliminated when subordinate trout were separated from their dominant tank-mate for 4 days of recovery. In contrast to the other indicators of muscle degradation, the activity of the 20S proteasome was reduced in subordinate trout regardless of when it was measured (i.e. interaction vs recovery), an unexpected observation that remains to be explained. With up to 30% of newly synthesized proteins rapidly degraded via the UPP (Wang et al. 2015), lower activity of the 20S proteasome in the muscle of subordinate fish might be linked to lower rates of protein synthesis, reflecting a reduction in the need for degradation of misfolded proteins. Collectively, the data support the hypothesis that reduced growth rates in subordinate trout result from reductions in protein synthesis coupled with increases in protein degradation, such that muscle protein accretion is reduced.

These findings are in broad agreement with the effects of chronic stress on muscle metabolism in juvenile fine flounder (*Paralichthys adspersus*). Four or seven weeks of exposure to crowding stress reduced growth, elevated circulating cortisol levels, and activated signalling pathways of the UPP, ALS and apoptosis in white muscle (Valenzuela et al. 2018). In particular, crowding stress increased transcript abundance of *murf1* as well as the abundance of ubiquitinated proteins, as in the present study. Interestingly, evidence of UPP activation was largely confined to four weeks of crowding stress, when cortisol levels were elevated, whereas evidence of autophagy was more prevalent at seven weeks of crowding stress, a time at which cortisol levels had returned to baseline values (Valenzuela et al. 2018). Chronic social stress, on the other hand, appeared to activate the UPP and ALS in parallel. In addition to the induction of proteolytic mechanisms, chronic crowding stress in fine flounder downregulated key elements of the growth hormone (GH)-insulin-like growth factor (IGF) system at both the endocrine level (i.e. GH, IGF-1 and their receptors and binding proteins), and at the cellular level in white muscle (signalling pathways). Within the protein kinase B/target of rapamycin (Akt/TOR) pathway, crowding stress decreased phosphorylation of Akt, TOR, the S6 ribosomal protein kinase (P70S6K), and the 4E-binding protein 1 (4E-BP1) (Valenzuela et al. 2018). Activation of this pathway and increased phosphorylation of these targets are implicated in stimulating protein synthesis (Seiliez et al. 2008; reviewed by Sadoul and Vijayan 2016), and therefore Akt/TOR downregulation in fine flounder experiencing chronic crowding stress was thought to contribute to growth reduction (Valenzuela et al. 2018). Investigation of the Akt/TOR pathway in white muscle of trout experiencing chronic social stress is warranted as a possible mechanism responsible for the inhibition of protein synthesis observed in the present study.

What factor(s) underlies the inhibition of protein synthesis and induction of proteolysis in white muscle of trout experiencing chronic social stress? A role for cortisol can be inferred from the observation that effects on muscle protein metabolism coincided with chronic elevation of cortisol levels in subordinate trout, and were eliminated in recovering subordinates in which cortisol had returned to baseline levels. Cortisol is involved in the regulation of various aspects of intermediary metabolism, including protein metabolism (Van der Boon et al. 1991). Recent work in zebrafish using ubiquitous glucocorticoid receptor (GR) and mineralocorticoid receptor (MR) knockout strains implicated elevated cortisol, acting through GR, in stimulating proteolysis (Faught and Vijayan 2019, 2020). Wild-type zebrafish treated with exogenous cortisol exhibited reduced growth and protein accumulation and increased transcript abundance of *murf1*, effects that were absent in GR-knockout fish (Faught and Vijayan 2020). In addition, GR-knockout fish had higher body mass, higher protein content and increased phosphorylation of eukaryotic initiation factor-4B (eIF4B), a marker for enhanced translation capacity (Faught and Vijayan 2019). These data suggest that elevated cortisol acts through GR to induce muscle proteolysis and inhibit muscle protein synthesis, resulting in the inhibition of growth. Similarly, elevated cortisol in subordinate fish may act on GR in white muscle to inhibit protein synthesis and induce proteolysis. Nevertheless, additional experiments are needed to establish a clear relationship among chronic social stress, elevated endogenous cortisol and alterations of protein metabolism.

The elevated rates of protein synthesis in the liver of subordinate trout were accompanied by increased transcript abundances of *cathepsin D* and *cathepsin L*, indicative of activation of the ALS. However, the transcript abundance of *mafbx* appeared to be significantly lower in subordinate fish, as was the abundance of polyubiquitinated proteins, suggesting lower activity in the UPP. These results are consistent with a dominant role for autophagy in hepatic proteolysis (Cassidy et al. 2016; Mortimore and Reeta Pösö 1986). Unlike white muscle, where markers of protein degradation were elevated in subordinate fish at 4 days of social interaction but not in recovering subordinates, sampling time had little effect on markers of protein degradation in the liver, suggesting more persistent effects of chronic social stress on liver protein metabolism.

Collectively, our results suggest that chronic social stress stimulates protein degradation in white muscle and protein synthesis in the liver. It is therefore likely that amino acids released by muscle proteolysis are used to support the higher rate of protein synthesis in the liver of fish experiencing chronic social stress. This pattern is consistent with the impact of chronic social stress in mobilizing carbohydrate and lipid energy reserves to support metabolism in the absence of incoming energy from food (Kostyniuk et al. 2018; DiBattista et al. 2006; Gilmour et al. 2012). These changes in protein metabolism are consistent with the chronic elevation of cortisol in socially stressed fish. Moreover, the return of cortisol to baseline levels in subordinate fish after 4 days of recovery from social interactions and the corresponding recovery of protein metabolism also argue for a key role of cortisol. Few studies to date have examined the cellular and molecular mechanisms underlying the well-known impact of chronic stress in reducing growth rates in fish. As with chronic crowding stress (Valenzuela et al. 2018), chronic social stress promoted muscle protein degradation via the ubiquitin–proteasome and autophagy pathways. A key finding of the present study is that inhibition of muscle protein synthesis also contributes to the growth suppressing effects of chronic stress.

## Supplementary Information

Below is the link to the electronic supplementary material.

Supplementary file1 (DOCX 22 KB)
