# Supplementary material for: Chronic social stress alters protein metabolism in juvenile rainbow trout, Oncorhynchus mykiss
Source: J Comp Physiol B. 2021 Mar 12;191(3):517–30. doi: 10.1007/s00360-021-01340-6 (PMC8043953; doi:10.1007/s00360-021-01340-6)
Supplement: Supplementary file 1 — Supplementary file1 (DOCX 22 KB) [file 360_2021_1340_MOESM1_ESM.docx]

**Supplementary material**

**Table S1**. A comparison of sham-treated and fasted sham rainbow trout (*Oncorhynchus mykiss*) at 4 d of “interaction”.

| **Variable** | **Sham-treated fish (*N* = 6)** | **Fasted sham fish (*N* = 5)** | ***P* value** |
| --- | --- | --- | --- |
| Plasma [cortisol] (ng mL^-1^) | 20.9 ± 9.3 | 10.0 ± 2.4 | 0.662† |
| SGR (% d^-1^) | -1.27 ± 0.25 | -1.16 ± 0.15 | 0.734 |
| **Liver** |  |  |  |
| *k_s_* (% d^-1^) | 10.0 ± 0.7 | 9.16 ± 0.41 | 0.327 |
| *cathepsin D* relative mRNA abundance | 1.00 ± 0.16 | 1.01 ± 0.14 | 0.961 |
| *cathepsin L* relative mRNA abundance | 1.00 ± 0.13 | 1.02 ± 0.15 | 0.937 |
| *mafbx* relative mRNA abundance | 1.00 ± 0.15 | 0.80 ± 0.13 | 0.354 |
| Polyubiquitinated protein relative abundance | 1.00 ± 0.07 | 0.91 ± 0.10 | 0.450 |
| **White muscle** |  |  |  |
| *k_s_* (% d^-1^) | 1.50 ± 0.13 | 1.21 ± 0.15 | 0.183 |
| *cathepsin D* relative mRNA abundance | 1.00 ± 0.22 | 1.37 ± 0.18 | 0.239 |
| *cathepsin L* relative mRNA abundance | 1.00 ± 0.26 | 1.39 ± 0.23 | 0.300 |
| *mafbx* relative mRNA abundance | 1.00 ± 0.21 | 1.14 ± 0.23 | 0.666 |
| *murf1* relative mRNA abundance | 1.00 ± 0.28 | 1.20 ± 0.09 | 0.177† |
| Polyubiquitinated protein relative abundance | 1.01 ± 0.06 | 0.88 ± 0.07 | 0.218 |

Values are means ± SEM. *P* values are for Student’s *t*-tests; † indicates rank sum test. Values for sham-treated trout are repeated from Table 2 and Figs. 2-5. mRNA and protein abundances are expressed relative to values for sham-treated fish.
